# Hypoxia-Activated Theragnostic Prodrugs (HATPs): Current State and Future Perspectives

**DOI:** 10.3390/pharmaceutics16040557

**Published:** 2024-04-19

**Authors:** Sankarprasad Bhuniya, Eirinaios I. Vrettos

**Affiliations:** 1 Centre for Interdisciplinary Sciences, JIS Institute of Advanced Studies and Research, JIS University, Kolkata 700091, India; spbhuniya@jisiasr.org; 2 Department of Chemical Biology and Therapeutics, St. Jude Children’s Research Hospital, Memphis, TN 38105, USA

**Keywords:** hypoxia-activated prodrugs (HAPs), hypoxia-activated theragnostic prodrugs (HATPs), cancer, tumor microenvironment, fluorescence, in vivo

## Abstract

Hypoxia is a significant feature of solid tumors and frequently poses a challenge to the effectiveness of tumor-targeted chemotherapeutics, thereby limiting their anticancer activity. Hypoxia-activated prodrugs represent a class of bio-reductive agents that can be selectively activated in hypoxic compartments to unleash the toxic warhead and thus, eliminate malignant tumor cells. However, their applicability can be further elevated by installing fluorescent modalities to yield hypoxia-activated theragnostic prodrugs (HATPs), which can be utilized for the simultaneous visualization and treatment of hypoxic tumor cells. The scope of this review is to summarize noteworthy advances in recent HATPs, highlight the challenges and opportunities for their further development, and discuss their potency to serve as personalized medicines in the future.

## 1. Introduction

Hypoxia constitutes a major contributing component of 90% of solid tumors and is triggered by the inadequate oxygen supply at a tissue level (typically < 1% O_2_), mainly due to problematic blood supply [1,2]. Hypoxia has been linked to cellular events that lead to poor cancer prognosis and therapeutic efficiency, as well as rapid tumor progression, angiogenesis, and metastasis [2,3,4,5]. It has, therefore, been a primary focus in the effort to develop effective treatment and imaging approaches to battle the menace of cancer [6,7,8,9,10]. Along these lines, hypoxic tumor regions are associated with elevated levels of nitroreductases (NTRs), enzymes that typically reduce aromatic nitro- and azo- moieties and, thus, this profusion renders them ideal targets for the spatiotemporal activation of nitro-/azo-bearing anticancer prodrugs [11]. This prevalent feature present in the tumor microenvironment is often utilized to generate imaging and therapeutic prodrugs (HAPs) that remain inactive under normoxic conditions but are selectively unleashed within hypoxic regions of solid tumors [7,11]. Evofosfamide (TH-302), which exhibits a 270-fold selective behavior for hypoxia over normoxia [12,13], constitutes a representative paradigm of nitro-bearing hypoxia-activated prodrugs that has reached up to Phase III clinical trials [14,15]. The combination of imaging and therapeutic modalities results in the formulation of hypoxia-activated theragnostic (therapy + diagnosis) prodrugs, designated HATPs. Although the design is modular, they typically consist of a moiety that interacts with hypoxia (‘trigger’), a fluorescent probe that can offer real-time visualization of malignant tumor cells, and a cytotoxic warhead that can effectively eliminate them. 

**Figure 1:**
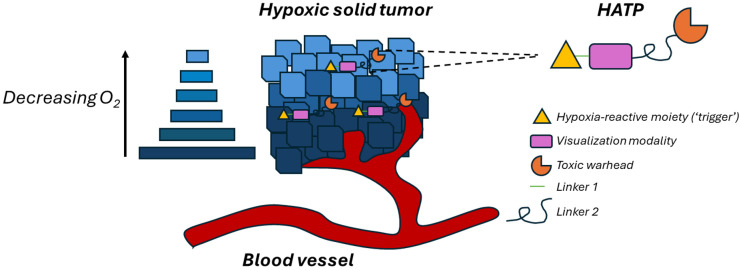
Schematic illustration of a typical hypoxia-activated theragnostic prodrug (HATP).

The four common individual components that constitute a hypoxia-activated theragnostic prodrug (HATP) are highlighted below and visualized in Figure 1:

(1) Toxic warhead: The drugs that are usually utilized to construct such prodrugs are typically highly toxic and are not able to distinguish malignant tumors from healthy cells [16]. They possess reactive characteristic groups that can be utilized as handles to attach additional pieces and construct the relevant prodrugs. In certain cases where the parent anticancer drug does not possess a conjugatable group, this can be installed after delicate design in strategic positions that will not interfere with its activity [17]. Representative examples are gemcitabine [18,19], 5-fluoro-2-deoxyuridine [20], paclitaxel [21], and nitrogen mustards [22]. Notably, certain fluorescent dyes can be utilized to simultaneously image and also treat tumors, typically due to characteristics that render them able to exert a photodynamic [23,24] or photothermal [25,26] effect.
(2) Visualization modality: Because of the many benefits that near-infrared (NIR) fluorescent dyes provide, they are generally preferred as the visualization modality. The utilized dyes can be uniquely selected for different applications based on their photophysical properties, solubility, reactive handles, and permeability. Such representative examples are various cyanines [22], Si-rhodamine [27], and Nile blue [28]. Nevertheless, there are instances where the fluorescent dye may emit fluorescence if the administered anticancer drug itself possesses inherent fluorescence, such as SN-38 or doxorubicin, allowing it to serve both functions concurrently [29].
(3) Hypoxia-reactive moiety (‘trigger’): Specific molecular groups can undergo reduction by NTRs, which are often overexpressed in the hypoxic regions of solid tumors characterized by low oxygen levels. For instance, the aromatic nitro group can be initially reduced to hydroxylamine and eventually to amine in the presence of NTRs [6]. Thus, nitro groups are inserted in strategic positions and stimulate drug release upon contact with NTRs, offering spatiotemporal drug release selectively within hypoxic sites [30]. A common nitroaromatic that is commonly implemented is 4-nitrobenzyl alcohol, which can release the toxic warhead via a 1,6-elimination cascade reaction when tethered together [30]. Other routinely utilized triggers are the diazobenzenes, azides, quinones, and N-oxides which are reduced under hypoxia to release the toxic warhead [31]. For instance, doxorubicin and MMAE (monomethyl auristatin E) classic anticancer agents can be converted to different HATPs that are selectively released in the hypoxic tumor microenvironment [6,32].
(4) Linkers: Various linkers are often utilized to connect all the different parts of an HATP. The linkers are generally selected based on their stability, presence of certain reactive handles and characteristic groups, and their length. Similar to PROTACs and other prodrugs, the effect of the linker is highly influential toward the overall bioactivity profile of the final HATP and should be selected in a prudent manner [16,33,34].
(5) Additional decorations: The utilization of additional modalities like tumor-homing elements (e.g., peptides [35,36,37], D-biotin [6,38]) or nano-formulations [39] can help ameliorate particular challenges. They are typically invoked to enhance tumor specificity by offering more precise delivery and the overall potency of the developed HATP.

The geometric assembly of the final HATP varies for each individual case, with the goal of providing the highest efficacy against a particular target. In this review, representative examples that consist of different components are presented, aiming to unveil the variability during the construction approach. Along these lines, in the following section, we aim to exemplify this modular design by pinpointing the uniqueness of each HATP that serves as an ideal approach for each specific objective.

## 2. Recent Representative Examples of HATPs

**Figure 2:**
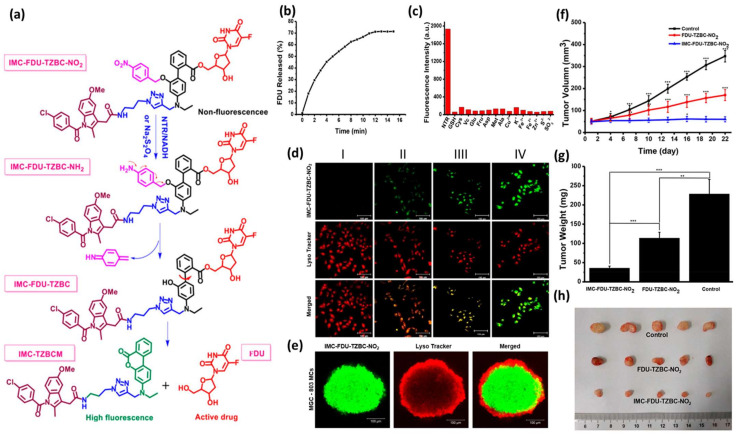
Mechanism of action and representative experimental results from IMC-FDU-TZBC-NO_2_**.** (**a**) Schematic representation of the concurrent drug (FDU) and dye (IMC-TZBCM) release to simultaneously treat and visualize hypoxic tumors. (**b**) Over-time FDU release from IMC-FDU-TZBC-NO_2_ (100 μM) after incubation with NTR (10 μg/mL). (**c**) Fluorescence intensity of IMC-FDU-TZBC-NO_2_ in PBS buffer during competition experiment using various species (10 μg/mL of NTR, 10 mM of GSH, Cys, Vc, Glu, Fru, Asp, Met, Ala, K^+^, Zn^2+^, Fe^3+^, Fe^2+^, Cu^2+^, S^2−^, SO_3_^2−^). (**d**) HeG-2 cells after treatment with IMC-FDU-TZBC-NO_2_ under normoxic (I) and hypoxic (II: 10% O_2_, III: 5% O_2_, IV: 1% O_2_) conditions for 12 h (scale bar 100 μm). (**e**) HepG-2 spheres after incubation with IMC-FDU-TZBC-NO_2_ and Lyso-Tracker red for 12 h (scale bar 100 μm). (**f**) Relative tumor volumes and (**g**) average changes of the tumor weight in 22 days of treatment (*n* = 5 and *, **, and *** means *p* < 0.05, < 0.01, or < 0.001, respectively). (**h**) Photographs of resected tumors of HepG-2 xenografted mice after treatment with IMC-FDU-TZBC-NO_2_, FDU-TZBC-NO_2_, and control—10 mg/kg per 3 days. Figure adapted with permission from [40].

Delicate rational design followed by classic chemical synthesis and meticulous biological evaluation has yielded several potent HATPs in the past years. Along these lines, Xiaoran Peng et al. reported the development of an HATP consisting of the toxic warhead 5-fluoro-2-deoxyuridine (FDU), a fluorescent dye (TZBCM), a tumor-homing element (indomethacin—IMC), and the hypoxia-reactive moiety 4-nitrobenzyl group which triggers the dissociation of the HATP in hypoxic compartments [40]. The elevated levels of NTRs associated with hypoxic conditions result in the simultaneous release of the drug (FDU) and the fluorescent reporter IMC-TZBCM (non-fluorescent before the release) selectively in the tumor microenvironment (Figure 2a). The authors initially wanted to confirm that NTRs interact with the trigger (reduction of the nitro group) which would consequently result in the drug release in adequate quantities to eliminate malignant tumor cells. Thus, they tested the effect of NTR on the dissociation rate of the parent HATP by quantifying the released FDU levels via HPLC (Figure 2b). After only 12 min of incubation, a plateau at 73% drug release was observed, proving its ability to rapidly release the chemotherapeutic in the presence of NTRs. Since complex biological systems contain multiple species that could interfere and possibly result in a premature drug release, the authors evaluated the activation of IMC-FDU-TZBC-NO_2_ via a competition experiment using NTR, saccharides, biothiols, amino acids, metal ions, and other reductants (Figure 2c). Notably, after separate incubation for 24 h they observed fluorescence intensity enhancement (λ_ex/em_ = 380/480 nm) only in the presence of NTR, providing additional proof of the selectivity of IMC-FDU-TZBC-NO_2_. To validate the selectivity of IMC-FDU-TZBC-NO_2_ towards hypoxia, they tested its fluorescence intensity in HepG-2 cells under different O_2_ conditions via confocal microscopy (Figure 1). Under normoxia (20% O_2_), IMC-FDU-TZBC-NO_2_ was non-reactive, exhibiting no fluorescence after cell incubation for 12 h at 37 °C (Figure 2(dI)). However, the fluorescence intensity was enhanced in a linear manner due to the reduction of the O_2_ levels (Figure 2(dII–dIV)). Confocal microscopy was further utilized to visualize the accumulation of IMC-FDU-TZBC-NO_2_ in HepG-2 3-D spheroids, demonstrating a strong fluorescence signal internally which is associated with hypoxia (Figure 2e). Therefore, the authors managed to validate the hypoxia-dependent nature of their presented HATP, which results in concurrent drug and fluorophore release. Finally, they evaluated the in vivo efficacy of IMC-FDU-TZBC-NO_2_, FDU-TZBC-NO_2_, and PBS (control) against HepG-2 xenografted mice after intravenous injection. IMC-FDU-TZBC-NO_2_ showed superior potency, demonstrating lower tumor volume and weight after 22 days of treatment (Figure 2f–h).

**Figure 3:**
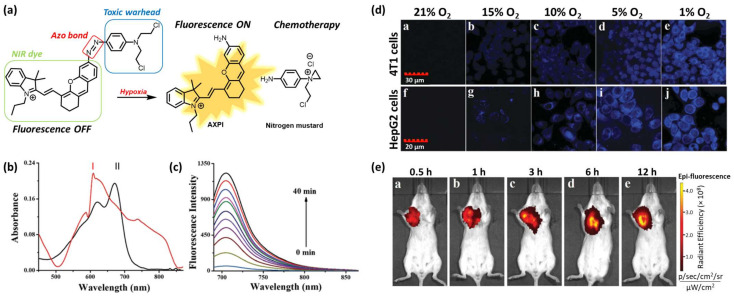
Mechanism of action and representative experimental results from a potent HATP. (**a**) Schematic representation of the concurrent drug (nitrogen mustard) and dye (AXPI) release for the simultaneous visualization and elimination of hypoxic tumors. (**b**) UV-Vis spectra of the HATP (10 μM) before (I) and after (II) chemical reduction with sodium dithionite (20 mM). (**c**) Fluorescence spectra of the HATP (10 μM) over time during the chemical reduction with sodium dithionite (20 mM). (**d**) Confocal imaging of the described HATP in 4T1 and HepG2 cells under increasing oxygen concentrations. (**e**) In vivo time-dependent fluorescence imaging of the HATP in BALB/c mice in vivo, after IV injection (500 mM in 100 mL PBS). Figure adapted with permission from [22].

Ning Ding et al. reported the development of an HATP consisting of a nitrogen mustard (toxic warhead), a NIR fluorophore (AXPI), and a hypoxia-reactive moiety (azo group) to enable the drug release selectively in hypoxic conditions (Figure 3a) [22]. The NIR dye was tethered to the anticancer agent via an azo bond, which rendered the fluorescence intensity quenched (“off” state). This bond can be reduced in the presence of elevated concentrations of NTRs within hypoxic compartments to result in the simultaneous drug (*N, N*-bis(2-chloroethyl)-1,4-benzenediamine) and fluorophore (AXPI) release, accompanied by high fluorescence intensity (“on” state). To validate that the reduction of the azo bond can trigger drug release and stimulate a fluorescence intensity enhancement, the authors performed a chemical reduction of their HATP using sodium dithionite (Na_2_S_2_O_4_). The HATP initially showed an absorption maximum at 600 nm in PBS (10 mM, pH 7.4, 1% DMSO), while after the chemical reduction, the maximum band was shifted to 670 nm due to the perturbation of the conjugation system (Figure 3b).

The low fluorescence intensity of the parent HATP (“off” state) was replaced by a high-intensity signal (“on” state) after the reduction of the azo bond (Figure 3c). The enrichment of the fluorescence intensity followed a time-dependent manner, reaching its maximum intensity after 40 min. This experiment furnished the first proof of concept before moving to more complex biologically related systems. Toward this aim, the authors evaluated its applicability in azoreductase-overexpressing cell lines 4T1 and HepG2 (Figure 3d). When both cell lines were treated with the parent HATP at normal O_2_ levels, the intracellular fluorescence intensity was negligible since the azo bond was unable to be reduced under these conditions. However, when they were treated under gradually decreasing O_2_ levels, the fluorescence intensity gradually increased, reaching the maximum intensity at the lowest level (1% O_2_). Thus, the hypoxia-selective nature of the presented HATP was further validated in cells. Finally, to demonstrate its selective hypoxia-triggered dissociation in the context of more complex biological systems, the authors proceeded to in vivo imaging experiments using 4T1 tumor-bearing BALB/c mice. As shown in Figure 3e, the fluorescence signal in the tumor area was substantially distinguishable from the other non-tumorous tissues over time, further validating the ability of the described HATP to selectively populate tumor cells and get activated by the enhanced levels of reductases.

**Figure 4:**
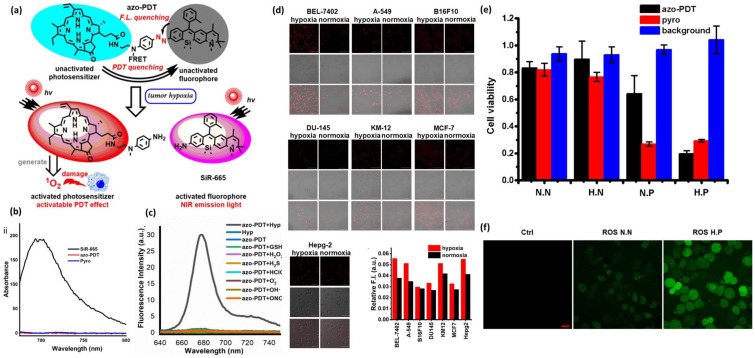
Mechanism of action and representative experimental results from azo-PDT. (**a**) Schematic illustration of its mechanism of action in hypoxic conditions. (**b**) Fluorescence spectra 5 µM of azo-PDT and its two individual components (SiR-665 and Pyro) in PBS. (**c**) Fluorescence intensity response of azo-PDT (5 μM) in the presence of various analytes in PBS (pH 7.4, 100 mM) containing 5% DMF as co-solvent at 37 °C with λ_ex_ 420 nm. (**d**) Comparison of the fluorescence intensity derived from azo-PDT (5 μM) after incubation with various cell lines under either hypoxia or normoxia (scale bar 100 μm). (**e**) Survivability of BEL-7402 cells after treatment with either azo-PDT or Pyro (2.5 μM) under normoxia or hypoxia, followed or not by photo-irradiation (150 W). N.N: Normoxia without photo-irradiation, H.N: Hypoxia without photo-irradiation, N.P: Normoxia with photo-irradiation, and H.P: Hypoxia with photo-irradiation. (**f**) Fluorescence confocal microscopy images of BEL-7402 cells after treatment with aza-PDT (2.5 μM), staining with 5 μM ROS indicator (2′, 7′-dichlorofluorescein diacetate) under normoxia without photo-irradiation (ROS N.N) or hypoxia with photo-irradiation for 20 min at 150 W (ROS H.P) (scale bar 20 μm). Figure adopted with permission from [27].

Similarly, Chang Wang et al. utilized the properties of the azo bond to construct an HATP, designated azo-PDT, that also included an NIR fluorophore as the visualization modality and pyropheophorbide α (photosensitizer) for the ablation of tumor cells via photodynamic therapy (PDT) [27]. The mechanism of action of azo-PDT is illustrated in Figure 4a. In brief, the utilized fluorophore, Si-Rhodamine (SiR-665), is an NIR-II-emitting fluorescent dye that can facilitate the real-time visualization of hypoxic tumors. Pyropheophorbide α is an analog of Photofrin (FDA-approved drug for PDT) that acts as a photosensitizer to eliminate malignant tumor cells on demand. Specifically, it absorbs energy through light to promote an electron to a single-state excited state, which is then interconverted to a triple-excited state. This triple state interacts with ^3^O_2_ to produce reactive oxygen species (ROS) to destroy the vicinal cells, an approach that is getting increasingly popular [41]. Azo-PDT was rationally designed to show low fluorescence intensity under normoxia (quench based on a FRET mechanism), but to result in high fluorescence intensity when it reaches hypoxic sites. To confirm this hypothesis, the authors performed a photophysical characterization of the HATP and its individual components. As expected, azo-PDT was associated with an extremely low fluorescence intensity due to a FRET quenching mechanism, while the plain NIR-II-emitting SiR-665 dye was highly emissive (Figure 4b). Furthermore, the authors tested if hypoxia can ‘activate’ the HATP in a selective manner. Thus, they performed a competition experiment that unveiled its hypoxia-selective switch-on nature. As shown in Figure 4c, the fluorescence intensity was solely elevated under hypoxia-mimicking conditions, providing hits for tumor-specific photodynamic efficiency.

Afterward, the hypoxia-enabled specificity of azo-PDT was evaluated in multiple live cells (BEL-7402, A-549, B16F10, DU-145, KM-12, MCF-7, Hepg-2) by using normoxic and hypoxic conditions. All utilized cell lines showed higher intracellular fluorescence intensity under hypoxia, which gradually increased over time following the reduction of the azo group, compared to normoxia (Figure 4d). BEL-7402 cells were then treated with azo-PDT under normoxia or hypoxia for 6 h and were then irradiated with LED light to trigger its photosensitizing effect. Azo-PDT showed a killing effect only under hypoxic conditions and only when it was accompanied by light irradiation, suggesting that its potency relies on both hypoxia and irradiation (Figure 4e). Finally, to confirm that the cytotoxicity relies on the production of ROS, they examined the cellular ROS levels by staining BEL-7402 cells with an ROS indicator (2′, 7′-dichlorofluorescein diacetate). In short, after incubation with azo-PDT, hypoxic cells were irradiated with LED light, but normoxic cells were not irradiated, and then both cell groups were stained with the ROS indicator. As shown in Figure 4f, only the hypoxic cells that were photo-irradiated for 20 min at 150 W showed high fluorescence derived from the presence of the indicator, proving that azo-PDT does induce the production of ROS in hypoxic conditions.

**Figure 5:**
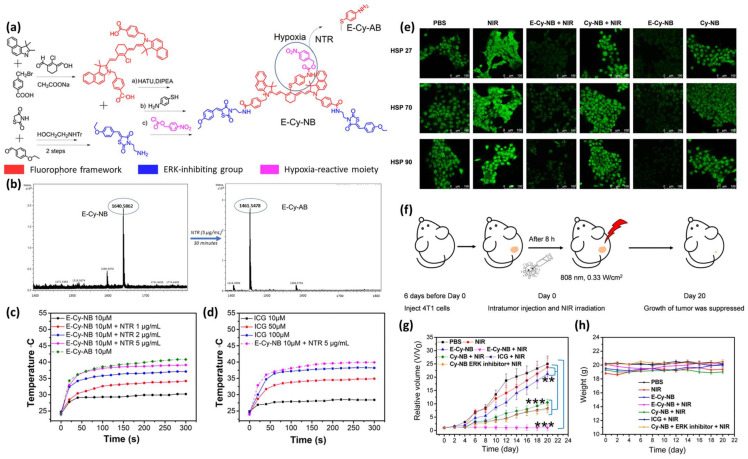
Mechanism of action and representative experimental results from E-Cy-NB. (**a**) Schematic representation of its structural components and its mechanism of action under hypoxic conditions. (**b**) HRMS spectra of E-Cy-NB before the addition of NTR (left panel) and E-Cy-AB that appears after the addition of NTR (right panel). (**c**) Temperature changes of E-Cy-NB (10 μM) after the addition of NTR (1–5 μg/mL), and the temperature of E-Cy-AB (10 μM) after NIR irradiation (808 nm). (**d**) Comparison of the temperatures of E-Cy-NB+NTR and ICG after NIR irradiation (808 nm). (**e**) Confocal microscopy pictures of 4T1 cells during the immunofluorescence analysis of three HSPs (scale bar 100 μm). (**f**) The protocol employed for the in vivo experiments against 4T1 tumor-bearing mice. (**g**) Tumor growth curves of mice after treatment with different combinations for 20 days (mean: SD, *n* = 5, ** *p* < 0.01, *** *p* < 0.001). (**h**) Body weight changes of each mouse group after 20 days of treatment. Figure adapted with permission from [26].

Xinhao Zhang et al. developed an NIR-emitting HATP, designated E-Cy-NB, that exerts its cytotoxic effect via photothermal therapy (PTT) in low temperatures [26]. E-Cy-NB can inhibit three types of heat shock proteins (HSPs) which protect cells from heat damage and are considered a crucial cause for PTT resistance. This agent consists of a cyanine dye (IR825) that serves both as the visualization and PTT modality (Figure 5a, red color), an ERK pathway inhibitor that reduces the expression of HSPs (Figure 5a, blue color), and a 4-nitrobenzyl group as the hypoxia-reactive moiety (Figure 5a, purple color). When E-Cy-NB exists under hypoxic conditions, the nitro group of the trigger is reduced and consequently cleaved to release E-Cy-AB that showed a higher photothermal effect (Figure 5a). Initially, the efficient cleavage of the nitro-containing trigger was evaluated via HRMS in the presence of NTR (5 μg/mL), to show the complete disappearance of E-Cy-NB (*m*/*z*: 1640.5862) and the formation of E-Cy-AB (*m*/*z*: 1461.5478) within 30 min (Figure 5b). Afterward, the authors aimed to validate its photothermal capability and compare it with a commercially available NIR dye that is typically utilized for PTT. As shown in Figure 5c, the temperature derived from E-Cy-AB was higher compared to E-Cy-NB, but the latter’s temperature equalized when irradiated and treated with 5 μg/mL NTR due to its conversion to E-Cy-AB. As shown in Figure 5d, E-Cy-AB (10 μM) was associated with higher photothermal temperature in comparison with the commercially available photothermal agent indocyanine green (ICG; 100 μM). To evaluate E-Cy-AB’s ability to inhibit HSPs, an immunofluorescence analysis in 4T1 cells was improvised. Using confocal microscopy the ability of E-Cy-NB to reduce the protein expression of three different HSPs when irradiated with NIR light was demonstrated (Figure 5e). The authors exploited a variety of tools to unveil the effect of E-Cy-NB on the ERK pathway and its ability to kill cancer cells in vitro. Finally, 4T1 breast tumor-bearing BALB/c mouse models were employed, and an appropriate protocol was utilized to demonstrate E-Cy-NB’s applicability in vivo (Figure 5f). The group treated with E-Cy-NB accompanied by NIR irradiation displayed complete tumor suppression, proving its ability to eliminate solid tumors via PTT (Figure 5g). Notably, the weights of the mice in all groups were not affected (Figure 5h).

**Figure 6:**
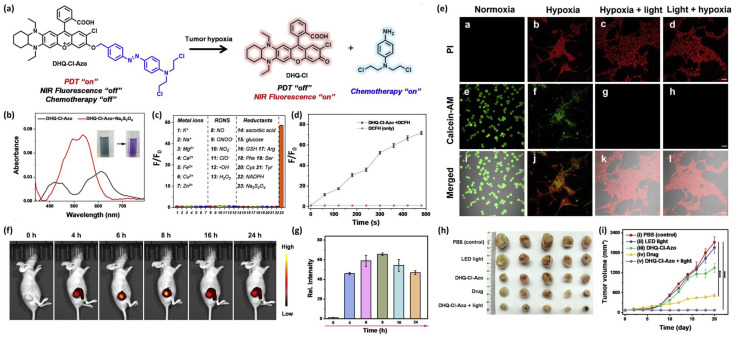
Mechanism of action and representative experimental results from DHQ-Cl-Azo. (**a**) Schematic representation of its structural components and its mechanism of action under hypoxic conditions. (**b**) UV-Vis spectra of DHQ-Cl-Azo (10 μM) before and after the addition of the reducing agent Na_2_S_2_O_4_. (**c**) Fluorescence enhancement of DHQ-Cl-Azo after the addition of various analytes: 100 μM metal ions (histograms 1–7), 100 μM RONS (histograms 8–13), 1 mM biological reductants (histograms 14–22), and Na_2_S_2_O_4_ (histogram 23). (**d**) In vitro PDT efficiency of DHQ-Cl-Azo over time via fluorescence enhancement after LED irradiation and the usage of an ROS indicator (DCFH). (**e**) Confocal microscopy pictures of dual-stained (PI and calcein-AM) 4T1 cells after treatment with DHQ-Cl-Azo (10 μM). Cells were cultured either under normoxia (panels **a**,**e**,**i**) or under hypoxia (panels **b**,**f**,**j**), or under hypoxia and LED irradiation (before or after) at 600 nm for 8 min (panels **c**,**g**,**k** or panels **d**,**h**,**l**) (scale bar 50 μm). (**f**) Images of 4T1-bearing mice during in vivo tumor imaging after intratumoral injection of DHQ-Cl-Azo. (**g**) Fluorescence intensity changes of the tumors. (**h**) Photographs of the tumors after 20 days of different therapeutic approaches and (**i**) comparison of the tumor volumes resected from the mice after different treatment approaches: PBS, 660 nm LED light irradiation, DHQ-Cl-Azo, drug (nitrogen mustard), and DHQ-Cl-Azo followed by 660 nm LED irradiation (*n* = 5); **** *p* < 0.0001). Figure adapted with permission from [42].

Jianhua Xiong et al. reported the development of an HATP, designated DHQ-Cl-Azo, which consists of an NIR-emitting fluorescent dye that is also utilized for PDT, a classic chemotherapeutic drug (nitrogen mustard) and an azo-group as the hypoxia-reactive moiety [42]. In the hypoxic environment of solid tumors, the azo-group is cleaved and both the NIR fluorophore and the anticancer drug are concurrently released. Upon their release, the anticancer drug eliminates the tumor cells via alkylation, while the NIR dye serves as the visualization modality (fluorescence enhancement) but can also produce singlet oxygen to eliminate the surrounding tissues via PDT (Figure 6a). The authors then wanted to validate the selective dissociation under reductive conditions and the efficiency of releasing singlet oxygen. Thus, they performed a chemical reduction of DHQ-Cl-Azo using Na_2_S_2_O_4_ and observed a significant change in the UV-Vis spectrum (Figure 6b) and fluorescence enhancement derived from the free NIR fluorophore, which was selective only for such reductive conditions (Figure 6c). The ability of DHQ-Cl-Azo to release ^1^O_2_ was verified in the buffer after 660 nm LED irradiation in the presence of DCFH (ROS indicator) and evaluation of the fluorescence enhancement (Figure 6d). The efficiency of DHQ-Cl-Azo to visualize and eliminate 4T1 cells was validated on live cells using the PI/calcein-AM dual staining and MTT under normoxic and hypoxic conditions. After treatment with DHQ-Cl-Azo, 4T1 cells cultured under normoxia remained alive, but those cultured under hypoxia died at a much higher pace (Figure 6e; panels a,b,e,f,i,j). Notably, hypoxic cells treated with DHQ-Cl-Azo and irradiated before or after with a 600 nm laser, presented high mortality rates (Figure 6e; panels c,d,g,h,k,l). Having proven DHQ-Cl-Azo’s applicability using in vitro settings, the authors proceeded to unveil its potency in vivo against 4T1 tumor-bearing mice. The tumor was associated with bright fluorescence, reaching its peak at 8 h post-injection, while being visible for at least 24 h (Figure 6f,g). The weights of the tumors after treatment with DHQ-Cl-Azo and followed by 600 nm irradiation were smaller compared to the other groups, showcasing its hypoxia-activated chemotherapeutic and PDT-associated potency (Figure 6h,i). The injection of DHQ-Cl-Azo followed by NIR irradiation proved to be a more effective approach in comparison with both the toxic drug (nitrogen mustard) and the plain DHQ-Cl-Azo without irradiation (Figure 6h,i).

**Figure 7:**
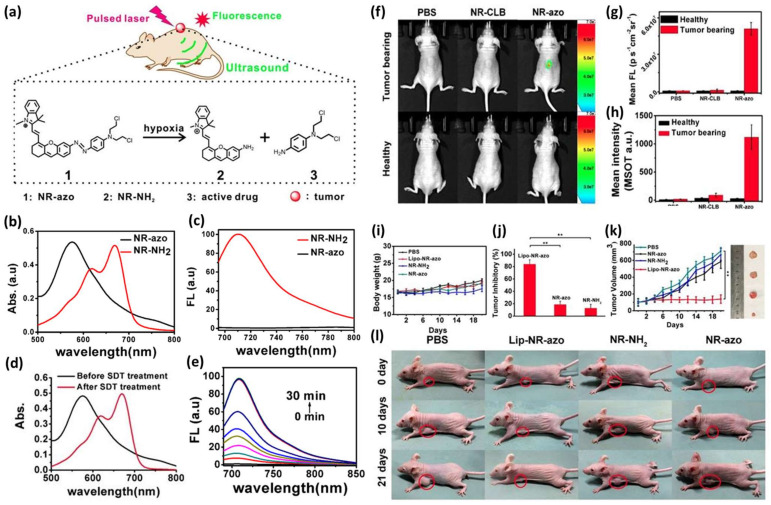
Mechanism of action and representative experimental results from NR-azo and Lipo-NR-azo. (**a**) Schematic representation of the components that compose NR-azo and the mechanism of simultaneous imaging and treatment of hypoxic tumors in mice. (**b**) UV-Vis spectra of NR-azo (10 μM) and NR-NH_2_ (10 μM) in PBS buffer. (**c**) Fluorescence spectra of NR-azo (10 μM) and NR-NH_2_ (10 μM) in PBS buffer using λ_ex_ = 680 nm. (**d**) UV-Vis spectra of NR-azo with and without the addition of NaS_2_O_4_ (100 μM). (**e**) Fluorescence spectra over time of NR-azo (10 μM) during the addition of NaS_2_O_4_ (100 μM). (**f**) Fluorescence images of HepG2-bearing mice (upper panel) and healthy mice (lower panel) after 30 min of intratumoral injection of PBS (200 μL), NR-CLB, or NR-azo. (**g**) Mean fluorescence intensity after injection of PBS, NR-CLB, or NR-azo. (**h**) Mean optoacoustic intensity after injection of PBS, NR-CLB, or NR-azo. (**i**) Mice body weight after treatment with PBS, Lipo-NR-azo, NR-NH_2_ and NR-azo. (**j**) Tumor inhibition rate (TIR) after treatment with Lipo-NR-azo, NR-azo, and NR-NH_2 over_ 21 days (** *p* < 0.01). (**k**) Tumor volume of tumor-bearing mice upon intravenous injection of PBS, NR-azo, NR-NH_2,_ or Lipo-NR-azo over 21 days (** *p* < 0.01). (**l**) Photographs of the mice at different time points after treatment with PBS, Lipo-NR-azo, NR-NH_2,_ and NR-azo. Figure adapted with permission from [43].

Jing Huang et al. reported the construction of a liposome-encapsulated HATP, named NR-azo, that can visualize tumor hypoxia via optoacoustic imaging and simultaneously inhibit tumor growth [43]. NR-azo consists of a xanthene-based NIR dye that serves as the visualization modality (optoacoustic imaging), an azo bond as the hypoxia-reactive moiety, and a nitrogen mustard as the toxic warhead (Figure 7a). NR-azo’s photophysical properties are predominantly altered compared to the free chromophore (NR-NH_2_) due to the installation of the electron-withdrawing azo bond. NR-azo has an absorption band at 575 nm, while NR-NH_2_ shows a peak at 680 nm (Figure 7b). Similarly, NR-NH_2_ displays a fluorescence peak at 710 nm, while NR-azo, when excited at the same wavelength, shows quench of the fluorescence intensity (Figure 7c). Based on these absorption and emission differentiations, the authors proved that the chemical reduction of the azo bond with Na_2_S_2_O_4_ results in the release of NR-NH_2_ (Figure 7d,e). After proving the optoacoustic and fluorescence potent effects of NR-azo in vitro, the authors proceeded to in vivo experiments. Initially, they validated its fluorescence and optoacoustic signals in HepG2-bearing mice. As shown in Figure 7f (upper panel) and Figure 7g, only the mice treated with NR-azo were associated with a strong fluorescence signal in the tumor region. The control group (PBS) and the plain NR-NH_2_ group (NR-CLB) showed no fluorescence. Similarly, no fluorescence was observed for the three groups in healthy mice (Figure 7f, bottom panel) highlighting the selective hypoxia-activated nature of NR-azo. Afterward, the authors encapsulated NR-azo into liposomes aiming to further enhance its tumor accumulation due to the EPR effect. The newly formulated polymeric agent, named lipo-NR-azo, was injected intravenously into HepG2-bearing mice (16.1 mg/kg) and its efficacy was compared to NR-NH_2_ (the plain fluorescent reporter; 8 mg/kg) and NR-azo (the agent without liposomes; (8 mg/kg). While maintaining their weight (Figure 7i), the mice in the group of Lipo-NR-azo were associated with a high tumor inhibitory rate (TIR) (Figure 7j) which resulted in the total inhibition of the tumor growth (Figure 7k,l) The tumor growth was highly suppressed. Therefore, Lipo-NR-azo proved to be an in vivo potent HATP that can simultaneously image and treat HepG2 tumors under hypoxia.

**Figure 8:**
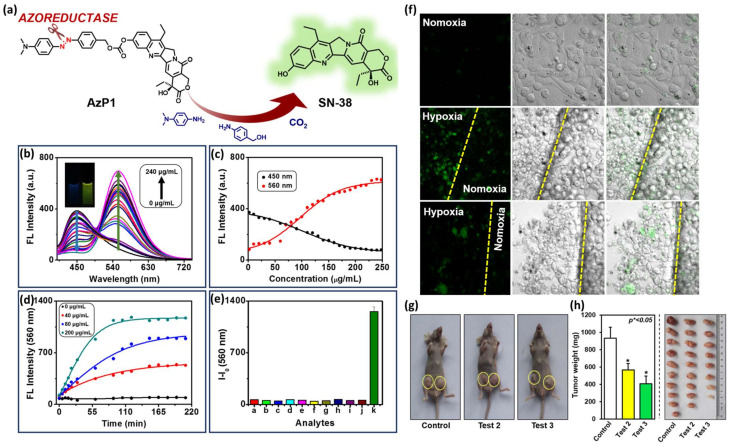
Mechanism of action and representative experimental results from AzP1. (**a**) Schematic representation of AzP1′s drug release mechanism under hypoxic conditions. (**b**) Fluorescence spectral changes of AzP1 (10 μM) after the gradual addition of increasing quantities of rat liver microsomes (0 μg/mL to 240 μg/mL). (**c**) Concentration-dependent fluorescence intensity changes of AzP1 (10 μM) regarding the peaks at 450 nm and 560 nm after the gradual addition of increasing quantities of rat liver microsomes (0 μg/mL to 240 μg/mL). (**d**) Time-dependent fluorescence intensity changes of AzP1 (10 μM) after the addition of specific concentrations of rat liver microsomes (0 μg/mL, 40 μg/mL 80 μg/mL, and 200 μg/mL). (**e**) Fluorescence intensity of AzP1 (10 μM) in the presence of several analytes (200 μM) a: ascorbic acid, b: GSH, c: NO, d: NO_2_^−^, e: H_2_O_2_, f: H_2_S, g: K_2_S_5_, h: tyrosinase, i: lipase, j: phosphatase, k: Na_2_S_2_O_4_. All fluorescence experiments were performed in DMSO-HEPES buffer (0.01 M, pH = 7.4) using λ_ex_ = 380 nm. (**f**) Confocal fluorescence microscopy images of HepG2 cells treated with AzP1 after having been cultured either under normoxia (21% O_2_) or hypoxia (3% O_2_) for 24 h (scale bar 20 μm). (**g**) Photographs of 4T1-bearing murine mice after treatment with either control or AzP1 at 10 μM (test 2) or AzP1 at 20 μM (test 3) for 21 days. (**h**) Tumor weight and size of the tumorous tissues after treatment with either control or AzP1 at 10 μM (test 2) or AzP1 at 20 μM (test 3) for 21 days. Figure adapted with permission from [44].

Ying Zhou et al. developed an HATP, named AzP1, that consists of an azo bond as the hypoxia-reactive moiety and SN-38 (FDA-approved antineoplastic agent) as both the toxic warhead and the visualization modality due to its inherent fluorescence properties [44]. Its fluorescence intensity is quenched but under hypoxic conditions, the azo bond reduction triggers the drug release that is also accompanied by high fluorescence intensity (Figure 8a). To prove this, the authors utilized rat liver microsomes, a reducing agent that is present under hypoxia, and monitored the changes in the fluorescence intensity. As shown in Figure 8b, AzP1’s fluorescence intensity maximum is gradually increased and red-shifted upon the addition of increasing amounts of rat liver microsomes (0–240 μg/mL). Specifically, the initial emission peak at 450 nm gradually disappeared and the peak at 560 nm increased both in a concentration- and time-dependent manner (Figure 8c and Figure 8d, respectively). As presented in Figure 8e, AzP1’s ratiometric response and selectivity for hypoxic environments were further validated by performing a fluorescence-based competition experiment with multiple substances present in actual biological samples: a. ascorbic acid, b. GSH, c. NO, d. NO_2_^−^, e. H_2_O_2_, f. H_2_S, g. K_2_S_5_, h. tyrosinase, i. lipase, j. phosphatase, and k. Na_2_SO_4_. After confirming that AzP1 exerts in vitro cytotoxic effects comparable to SN38, the authors validated AzP1’s selective behavior in hypoxic over normoxic conditions. As shown in Figure 8f, AzP1 is highly selective for HepG2 cells cultured under hypoxic over normoxic conditions, rendering it superior to plain SN38 which is correlated by a dominant uncontrolled toxicity. Finally, AzP1’s efficacy was explored in vivo against 4T1-xenografted murine mice (Figure 8g,h). The animals were tested with either control or AzP1 10 μM (test 2) or AzP1 20 μM (test 3) via an intravenous injection 2/week for 3 weeks to prove AzP1 potency in diminishing the tumor growth.

**Figure 9:**
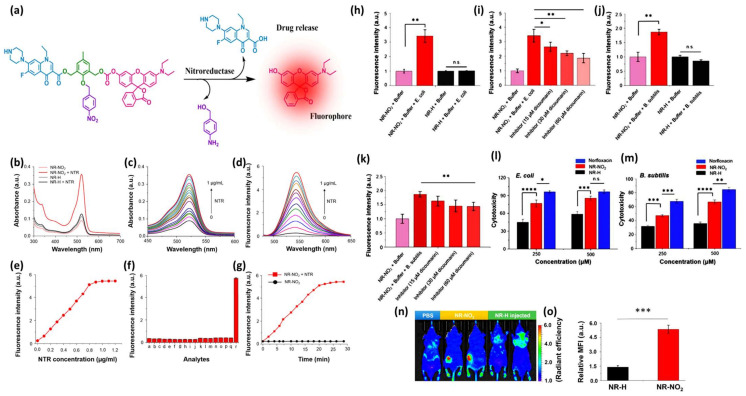
Mechanism of action and representative experimental results from NR-NO_2_. (**a**) Schematic representation of NR-NO_2_’s drug release mechanism under hypoxic conditions. (**b**) UV-Vis spectra of 10 μM of NR-NO_2_ (HATP) or NR-H (control) with and without the presence of NTR. (**c**) UV-Vis spectral changes of NR-NO_2_ upon the addition of increasing concentrations of NTR (0–1 μg/mL). (**d**) Fluorescence spectral changes of NR-NO_2_ upon the addition of increasing concentrations of NTR (0–1 μg/mL). (**e**) Fluorescence intensity of NR-NO_2_ (10 μM) when incubated for 20 min at 37 °C with different NTR concentrations (λ_ex_/λ_em_ = 525/545 nm). (**f**) Fluorescence intensity of NR-NO_2_ (10 μM) upon the addition of various biological-related substances: a. blank (NR-NO_2_ + NADH in PBS) and serum-containing b. KCl (150 mM), c. MgCl_2_ (2.5 mM), d. CaCl_2_ (2.5 mM), e. glucose (10 mM), f. vitamin C (1 mM), g. vitamin B6 (1 mM), h. HAS (100 μM), i. H_2_O_2_ (10 μM), j. lipase (200 U/L), k. pepsin (100 μg/L), l. trypsin (200 ng/mL), m. glutamic acid (1 mM), n. arginine (1 mM), o. serine (1 mM), p. glutathione (5 mM), q. cysteine (1 mM), and r. NTR (1 μg/mL). (**g**) Fluorescence intensity changes of NR-NO_2_ (10 μM) over time with (red line) and without (black line) the presence of NTR (0.5 μg/mL). (**h**) Relative fluorescence intensity of *E. coli* lysate after treatment with NR-NO_2_. (**i**) Consequent changes in the fluorescence intensity of *E. coli* lysate treated with NR-NO_2_, in the presence of a nitroreductase inhibitor (15, 30, and 60 μM dicoumarol). (**j**) Relative fluorescence intensity of *B. subtilis* lysate after treatment with NR-NO_2_. (**k**) Consequent changes in the fluorescence intensity of *B. subtilis* lysate treated with NR-NO_2_, in the presence of a nitroreductase inhibitor (15, 30, and 60 μM dicoumarol). (**l**) Cytotoxicity graphs of NR-NO_2_, NR-H, or norfloxacin (250 and 500 μM) after 24 h incubation with *E. coli*. (**m**) Cytotoxicity graphs of NR-NO_2_, NR-H, or norfloxacin (250 and 500 μM) after 24 h incubation with *B. subtilis*. (**n**) In vivo optical imaging photographs of A549 tumor-bearing mice after treatment with PBS, NR-NO_2,_ or NR-H. (**o**) Relative fluorescence intensity within the tumor region after treatment with NR-NO_2_ or NR-H. In all the cases * *p* < 0.05; ** *p* < 0.01; *** *p* < 0.001; and **** *p* < 0.0001; n.s.: non significant. Figure adapted with permission from [45].

Utilizing a slightly different approach than the above-mentioned examples, Sanu Karan et al. published an HATP, named NR-NO_2_, that can visualize the solid tumor and treat bacterial infections [45]. The idea was predicated on the notion that bacteria are intimately linked to cancer resistance and metastasis [46,47,48]. Specifically, NR-NO_2_ includes a central core molecule that is decorated with a hypoxia-reactive moiety, a fluorescent probe, and an antibiotic agent. NR-NO_2_ stays initially inactive (fluorescence is quenched), but it gets activated with the concurrent drug and fluorophore release upon interaction with nitroreductases (Figure 9a). Initially, the authors aimed to confirm NR-NO_2_’s ability to interact with NTR and unleash the visualization and treatment modalities. NR-NO_2_’s (5 μM) UV-Vis maximum absorption (522 nm) increased dramatically upon interaction with NTR (0.01 mg/mL) (Figure 9b,c). In a similar fashion, the fluorescence intensity of the main band (545 nm) gradually increased up to 23 times after the addition of NTR (0 to 1.2 μg/mL) (Figure 9d), via a linear relationship (Figure 9e). Through a fluorescence-based competition experiment by using other biological-related substances (anions and cations, reducing agents, ROS, enzymes, and amino acids), it was proved that NR-NO_2_ is selective toward nitroreductase (Figure 9f) and can evade a premature or uncontrolled drug release. It was also proved that NR-NO_2_ unleashes its components (fluorophore and drug) quantitively after 20 min of interaction with NTR (Figure 9g).

Then, NR-NO_2_’s ability to interact with microbial nitroreductases in live microorganisms was evaluated against *E. coli* (Gram-negative) and *B. subtilis* (Gram-positive). *E. coli* bacteria exhibited a ~3.5-fold fluorescence enhancement compared with the two controls (Figure 9h), which could be inhibited if a reductase inhibitor (dicoumarol—15 to 60 μM) was added (Figure 9i), pinpointed its selectivity to unleash the drug. Notably, an identical behavior was also observed when the same methodology was applied to *B. subtilis* (Figure 9j,k). Simultaneously, NR-NO_2_ proved to be bactericidal against both bacterial strains, displaying activity like that exerted by the classic antibacterial agent Norfloxacin (Figure 9l,m). Finally, the authors provide NR-NO_2_’s ability to visualize solid tumors in vivo in A549 tumor-bearing mice, where they observed that its fluorescence intensity around the tumor region was ~4 times higher than the control (Figure 9n,o). Therefore, NR-NO_2_ could pave the way for the development of HATPs that function as simultaneous tumor diagnostic and bacterial infection therapeutic modalities.

## 3. Discussion

The abovementioned paradigms showcase the selective hypoxia-targeted simultaneous diagnosis and therapy approach that is continuously gaining attention for the elimination of demanding solid tumors. The construction of HATPs mandates a delicate design approach but is also characterized by high modularity. A vast number of warheads, dyes, linkers, triggers, tumor-homing elements, etc., can be compounded in different ways to produce the optimal therapeutic agents for every specific case, thus, rendering it as leading contestant in the field of personalized medicine.

As explicitly shown above, the utilized therapeutic modality usually varies between classic toxic anticancer agents, photodynamic, and photothermal therapy according to the particular requirements. For instance, photodynamic therapy (PDT) is an intriguing approach often invoked to effectively battle the menace of cancer. However, its efficacy is limited within the hypoxic environment of solid tumors, thus diminishing the potency of the developed HATPs [24,49]. Therefore, special attention needs to be paid and specific warheads should be selected according to the literature. Notably, An et al. presented a strategy to effectively surpass this limitation based on the biotinylation of the utilized PDT modalities [24]. Turan et al. have shown that the installation of 2-pyridone could be useful in fractional PDT (light irradiation in intervals) by being converted to an endoperoxide during the irradiation step, which can then release singlet oxygen during the dark step [50]. Lv et al. developed a potent photosensitizer based on a Ruthenium-based modified coumarin that showed enhanced PDT effect due to the charge transfer between the excited photosensitizer and an adjacent substrate, forming a reactive radical ion (type I PDT) to eliminate solid tumors [51]. This approach could act as the springboard for the development of similar charge-optimized photosensitizers as effective treatment options against hypoxic tumors.

In a similar fashion, the utilized imaging modality can be selected among a wide pool of available fluorescent dyes to serve the purpose of solid tumor visualization. The copious number of the accessible probes (e.g., coumarins, rhodamines, fluoresceins, cyanines, squaraines, etc.) or drugs that are endowed with autofluorescence (e.g., SN-38, doxorubicin, etc.) facilitate the development of personalized HATPs. The design is further smoothed by the continuously growing number of existing linkers in the era of PROTACs [33,52], able to tether the individual components regardless of their characteristic groups.

Currently, hypoxia-activated theragnostic prodrugs (HATPs) play a significant role in targeting hypoxic tissues. However, due to the complex and heterogeneous microenvironment of tumors and the cytotoxic side effects of drugs, only a few drugs can successfully pass the rigorous tests necessary for drug discovery and application in clinical chemotherapy. At present, many candidate drugs lack sufficient hypoxia selectivity to progress through clinical trials. Furthermore, comprehensive systematic analysis, including various model studies such as stochastic population dynamics processes, is needed to understand the effective drug concentration and oxygen distribution within the heterogeneous microenvironment.

To advance this strategy to the clinical trial phase, high-throughput methodologies are required to screen ideal candidates for clinical phases. Additionally, a deeper understanding of the interactions between HATPs and targeted cellular microenvironmental components is essential. This understanding can provide insights into the suitable conditions for hypoxia-activated molecular structure transformations. Currently, detailed information regarding the pharmacokinetics and pharmacodynamics of reported HATPs approaches is not well-documented. Although HATPs show great promise to serve as future effective treatments against hypoxic tumors, they are also associated with certain impediments that need to be surpassed to aid their advancement to the market.

## 4. Future Perspective

Hypoxia-activated therapeutic and consequently theragnostic prodrugs (HAPs and HATPs, respectively) hold significant potential to revolutionize cancer prognosis and treatment, allowing for personalized therapy in the future [53]. Their development is promising and multifaceted, presenting several critical avenues for exploration and advancement in cancer therapeutics.

Firstly, there is a need for continued research into the design and synthesis of novel HATPs with enhanced specificity and efficacy. This entails identifying new molecular targets and developing innovative chemical structures that can selectively activate within hypoxic tumor microenvironments while minimizing off-target effects in normal tissues. Advanced drug delivery systems, such as nanoparticles or liposomes, may also be integrated to improve the pharmacokinetic profile and tumor accumulation of hypoxia-activated prodrugs.

Secondly, the development of imaging techniques for real-time monitoring of hypoxia within tumors is crucial for guiding therapeutic interventions and assessing treatment responses. Incorporating diagnostic elements into HAPs, such as fluorescent or radioactive tags, allows for non-invasive imaging of drug distribution, metabolism, and therapeutic efficacy. Furthermore, the integration of molecular imaging modalities into HAPs, such as positron emission tomography (PET) or magnetic resonance imaging (MRI), enables precise localization and quantification of hypoxic regions within tumors.

Moreover, exploiting synergistic treatment approaches through combination therapy strategies holds immense potential in improving the therapeutic outcomes for cancer patients. Combinatorial regimens involving HATPs in conjunction with conventional chemotherapeutic agents, radiotherapy, immunotherapy, or targeted therapies can enhance tumor cell killing, overcome drug resistance, and minimize adverse effects. Rational drug design approaches and preclinical studies are needed to identify optimal drug combinations and treatment schedules for different cancer types and patient populations.

Furthermore, advancements in personalized medicine and biomarker discovery offer opportunities for patient stratification and tailored treatment strategies in the context of hypoxia-activated theragnostic agents [54]. Their modular assembly may enable the sculpting of agents that could be precisely tailored to the tumor characteristics of particular patients, resulting in superior effectiveness. Additionally, identifying predictive biomarkers associated with tumor hypoxia and drug response enables the selection of patients who are most likely to benefit from hypoxia-activated prodrug therapy. This necessitates the development of robust diagnostic assays and genomic profiling techniques for assessing hypoxia biomarkers and predicting treatment outcomes.

Lastly, translating preclinical findings into clinical practice requires rigorous evaluation through well-designed clinical trials. Conducting Phase I/II studies to assess the safety, pharmacokinetics, and preliminary efficacy of hypoxia-activated prodrugs in cancer patients is essential for establishing their therapeutic potential and optimizing treatment protocols. Collaborative efforts between academia, industry, and regulatory agencies are essential for expediting the clinical development and regulatory approval of HATPs, ultimately bringing innovative cancer treatments to patients in need.
